# Communication Behavior Changes Between Patients With Diabetes and Healthcare Providers Over 9 Years: Retrospective Cohort Study

**DOI:** 10.2196/17186

**Published:** 2020-08-11

**Authors:** Arriel Benis, Refael Barak Barkan, Tomer Sela, Nissim Harel

**Affiliations:** 1 Faculty of Technology Management Holon Institute of Technology Holon Israel; 2 Clalit Research Institute Clalit Health Services Tel-Aviv Israel; 3 Faculty of Sciences Holon Institute of Technology Holon Israel; 4 Online Division Clalit Health Services Tel-Aviv Israel

**Keywords:** population characteristics, eHealth, mHealth, consumer health informatics, delivery of health care, machine learning, clustering, quality of health care, point-of-care systems, physician-patient relations

## Abstract

**Background:**

Health organizations and patients interact over different communication channels and are harnessing digital communications for this purpose. Assisting health organizations to improve, adapt, and introduce new patient–health care practitioner communication channels (such as patient portals, mobile apps, and text messaging) enhances health care services access.

**Objective:**

This retrospective data study aims to assist health care administrators and policy makers to improve and personalize communication between patients and health care professionals by expanding the capabilities of current communication channels and introducing new ones. Our main hypothesis is that patient follow-up and clinical outcomes are influenced by their preferred communication channels with the health care organization.

**Methods:**

This study analyzes data stored in electronic medical records and logs documenting access to various communication channels between patients and a health organization (Clalit Health Services, Israel). Data were collected between 2008 and 2016 from records of 311,168 patients diagnosed with diabetes, aged 21 years and over, members of Clalit at least since 2007, and still alive in 2016. The analysis consisted of characterizing the use profiles of communication channels over time and used clustering for discretization purposes and patient profile building and then a hierarchical clustering and heatmaps to visualize the different communication profiles.

**Results:**

A total of 13 profiles of patients were identified and characterized. We have shown how the communication channels provided by the health organization influence the communication behavior of patients. We observed how different patients respond differently to technological means of communication and change or don’t change their communication patterns with the health care organization based on the communication channels available to them.

**Conclusions:**

Identifying the channels of communication within the health organization and which are preferred by each patient creates an opportunity to convey messages adapted to the patient in the most appropriate way. The greater the likelihood that the therapeutic message is received by the patient, the greater the patient's response and proactiveness to the treatment will be.

**International Registered Report Identifier (IRRID):**

RR2-10.2196/10734

## Introduction

### Background

Communications between patients and health care professionals are based on a range of communication channels [1-10] and influenced by cultural factors [11-13]. Traditional channels supporting these interactions are face-to-face visits and phone calls. Health management organizations (HMOs) are capitalizing on the digital revolution [8,14] and innovating and providing patients with new digital tools [15]. Their goal is to provide patients with alternative ways for asking, getting, and sharing health-related information and knowledge [2-6,9,10,16].

Interactions between patients and health care professionals in an HMO must be analyzed over time to better understand the potential impacts of technological changes. Data mining and machine learning methodologies are used in the analysis of a large amount of data. Several techniques can be used to define or redefine clusters of patients based on sociodemographics and biological and clinical data [17,18]. We are not aware of an attempt to cluster patients based on communication, sociodemographic, and bioclinical characteristics, let alone at a large scale involving data from hundreds of thousands of patients collected for almost a decade. In this paper, we are disclosing the results of this kind of approach [19].

### Aims and Objectives

This retrospective data study aims at assisting health care administrators in defining and developing new communication channels and policy makers in improving and personalizing communication between patients and health care professionals (eg, physicians and nurses). By expanding the capabilities of currently available communication channels and introducing new channels, we hope to help policy makers enhance the accessibility of health care professionals and organizations and improve the quality of patient follow-up and treatment adherence and the overall patient experience with HMO services [13,19-22].

This work characterizes the use profiles of chronic patients with the communication channels available at Clalit Health Services, a large HMO in Israel, between 2008 and 2016. The use profiles are then associated with sociodemographic and medical patient profiles.

The leading objective of this analysis is to propose new ways to promote the use of the most appropriate communication channels based on the patient profile. An additional objective is to recommend sociological and technological ways that should be developed for increasing the quality and effectiveness of the patient–health care professional communication and interaction.

### Hypotheses

This retrospective research is led by 3 hypotheses:

- Preferred communication channels of a patient with the health care professional in an HMO influence follow-up and clinical outcomes.
- Adoption of a new communication channel by a patient is affected by their sociodemographic and clinical profile.
- Introducing a new communication channel impacts the use of existing communication channels.

This research focuses on quantifying these behaviors. The goal is to identify sociodemographic and bioclinical attributes affecting engagement with newly launched communication channels. This research characterizes changes in the use of existing communication channels once a new communication channel is introduced.

## Methods

The study design including details about material and methods has been described and published elsewhere [19]. Ethical approval for the study was granted by the Clalit ethical committee.

### Material

Data were extracted from Clalit electronic medical records (EMRs), which include documented access to various communication channels between patients and Clalit. Clalit is the largest Israeli HMO, with 4.53 million insured members (53% of the Israeli population) in 2016. Since 1998, Clalit’s EMRs have been stored in a data warehouse [19,23,24].

**Figure 1:**
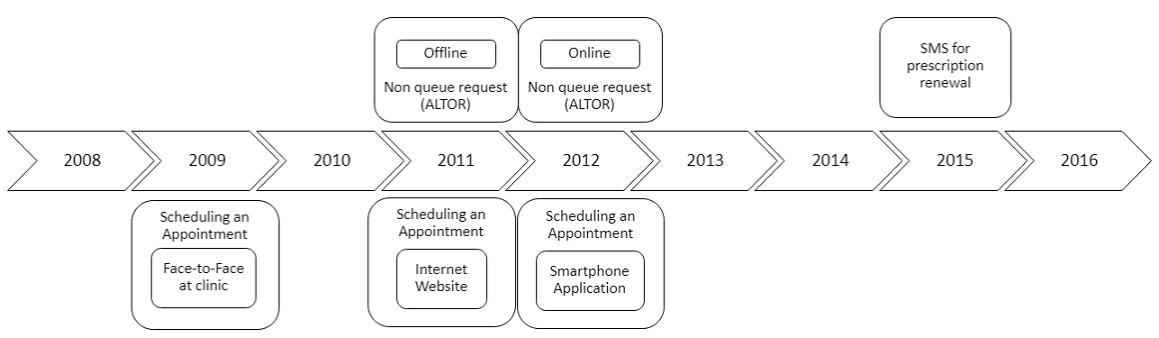
Communication channel introduction over time.

The period of time investigated in this research allowed us to analyze the launch of new communication channels such as a website, mobile apps, and text messaging (short message service [SMS]) system (Figure 1) and identify communication behavior changes as functions of time and the introduction of new communication channels.

The cohort consisted of patients aged 21 years and older, diagnosed with diabetes, members of Clalit for at least 1 year before 2008, and still alive in 2016 [25-27].

### Analysis Process

#### Overview

This research used the knowledge discovery in databases (KDD) framework [28-30]. Our study analyzes communication channel use over a 9-year period wherein Clalit introduced and changed the methods of interactions between patients, health care professionals, and administrative staff. We identified the sociodemographic and bioclinical characteristics for each communication profile and qualitatively evaluated the influence of the profile on patient engagement and follow-up quality.

We ran 1-dimensional and multidimensional statistical tools and different data mining algorithms, which were used during the data cleansing step [31,32]. The data extraction, preprocessing, data mining, and information visualization are briefly described below. Details have been published elsewhere [19].

#### Data Extraction

Data extracted from the Clalit data warehouse for each patient included sociodemographic [19] and bioclinical [25,26,33-37] data and contacts with the HMO using communication channels.

#### Data Preprocessing

Cleansing of extracted data reduced noise by detecting and removing or correcting outliers [38,39]. An outlier is a data measurement that is inconsistent with other historical measurement data of the same individual. For some measurements (eg, BMI), specific algorithms have been developed in-house by Clalit. In the absence of these algorithms, statistical approaches and machine learning algorithms were used [40-44].

Several machine learning algorithms require data reformulation to support data categorization or grouping numerical, categorical, or textual data [41,45-48]. For some attributes that don’t have predefined scales, we used the k-means clustering algorithm to discretize the data into 6 groups: very small, small, small-to-moderate, moderate, moderate-to-large, and large. The cluster bounds were validated by a domain expert (Table 1).

**Table 1:** Gradient reformulation and ranges of values related to each resource consumption level.

Characteristic		Gradient reformulation						
		N/A^a^	No (very small)	Small	Small to moderate	Moderate	Moderate to large	Large
**Contact with health care provider**								
	Physician consultation	N/A	0	1-7	8-19	20-28	29-51	52+
	Nurse consultation	N/A	0	1-3	4-5	6-8	9-14	15+
	Hospitalization	N/A	0	1	2	3	4-5	6+
	ED^b^ visit	N/A	0	1	2	3	4	5+
**Scheduling an appointment**								
	Face-to-face at clinic	N/A	0	1	2	3-4	5-8	9+
	Call to clinic or call center	N/A	0	1-2	3-5	6-9	10-15	16+
	Smartphone app	N/A	0	1	2	3-4	5-8	9+
	Internet website	N/A	0	1	2-3	4-6	7-10	11+
**Nonqueue request**								
	Online	N/A	0	1-3	4-12	13-24	25-45	46+
	Offline that must be done online	N/A	0	1-17	18-44	45-74	75-113	114+
	Offline	N/A	0	1-17	18-44	45-74	75-113	114+
**Pharmacy**								
	Overall recorded visits	N/A	0	1-4	5-12	13-26	27-42	43+
**SMS^c^ for prescription renewal**								
	Proposition sent by HMO^d^ to patient	N/A	0	1	2	3	4	5+
	Approval sent by patient to HMO	N/A	0	1	2	3	4	5+

^a^N/A: not applicable.

^b^ED: emergency department.

^c^SMS: short message service.

^d^HMO: health management organization.

#### Data Mining and Information Visualization

As we don’t have prior knowledge on communication channel use, we used unsupervised learning algorithms, mainly k-means and hierarchical clustering [47-56] combined with the Ray-Turi criterion [49].

To investigate communication patterns over time, we built heatmaps for each year between 2008 and 2016 based on the previously generated hierarchical clustering of 2016 data. Furthermore, we concatenated the communication profile of each discovered patients’ clusters over the years [56]. This visualization helps identify changes in communication profiles for each cluster.

All computations described above were performed using R 3.3.1 (R Foundation for Statistical Computing) with the following packages: data.table [57] (computing efficiency given the large data size), cluster [58] (k-means and hierarchical clustering), and gplots [59] (drawing heatmaps and hierarchical clustering dendrograms).

## Results

### Overview

**Figure 2:**
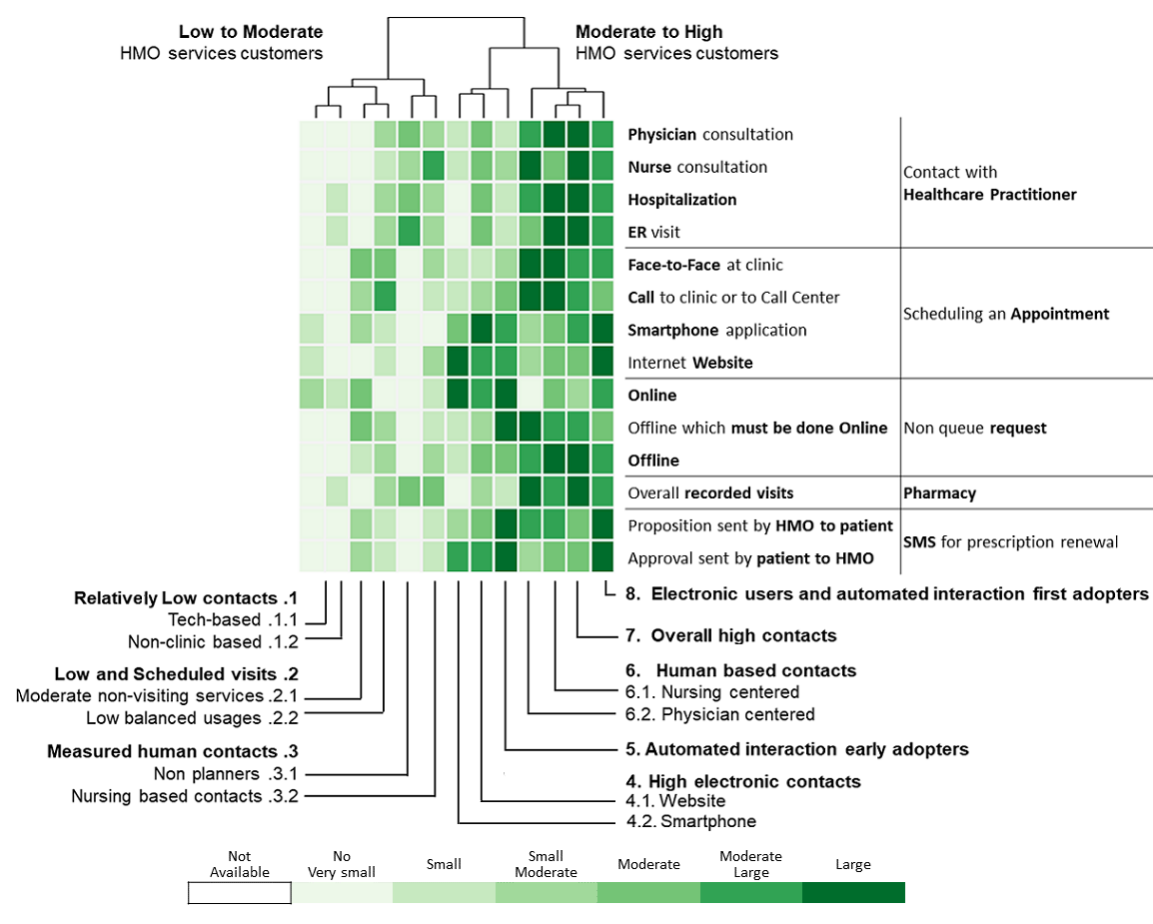
Communication patterns in 2016 of 311,168 patients with diabetes (members of the Clalit Health Services). HMO: health management organization.

A total of 311,168 individuals were included in the study cohort. As means of communication with health care professionals have changed throughout the research period, we chose 2016 as the base year to which communication behavior is compared because during this year, health care customers were offered the most diverse communication channels. Applying the methodology described above to the 2016 data revealed 13 communication profiles. The resulting heatmap is presented in Figure 2. Two main types of communication behaviors are evident from the figure. The first main cluster consists of 6 communication profiles describing low-to-moderate health care services consumption. The second one consists of 7 communication profiles describing moderate-to-high health care services consumption.

All differences between the sociodemographic, biological, clinical (Tables 2 and 3), and communication characteristics between the overall population and each one of the clusters were statistically significant (*P*<.001).

**Table 2:** Sociodemographic, clinical, and biological measurements data summary for patients with diabetes, in 2016, having a low-to-moderate health care services consumption.

Characteristic			Overall (n=311,168)		Relatively low contacts				Low and scheduled visits				Measured human contacts		
					Tech-based (n=35,719)		Non–clinic-based (n=34,060)		Moderate nonvisiting services (n=49,540)		Low balance use (n=41,735)		Nonplanners (n=22,275)		Nursing-based contacts (n=29,197)
**Gender, n (%)**															
	Female	156,269 (50.2)		15,669 (43.9)		17,173 (50.4)		24,609 (49.7)		22,525 (54.0)		11,264 (50.6)		15,463 (53.0)	
	Male	154,899 (49.8)		20,050 (56.1)		16,887 (49.6)		24,931 (50.3)		19,210 (46.0)		11,011 (49.4)		13,734 (47.0)	
Age in years, median (IQR^a^)			68 (60-77)		60 (51-68)		69 (61-79)		67 (60-76)		70 (62-78)		68 (60-76)		70 (62-79)
**Immigrant, n (%)**															
	No	152,533 (49.0)		23,602 (66.1)		18,712 (54.9)		21,710 (43.8)		15,921 (38.1)		14,504 (65.1)		16,085 (55.1)	
	Yes	158,635 (51.0)		12,117 (33.9)		15,348 (45.1)		27,830 (56.2)		25,814 (61.9)		7771 (34.9)		13,112 (44.9)	
**Ethnicity, n (%)**															
	General	242,022 (77.8)		24,232 (67.8)		23,486 (69.0)		41,613 (84.0)		34,218 (82.0)		12,456 (55.9)		19,899 (68.2)	
	Arab	60,619 (19.5)		10,534 (29.5)		9884 (29.0)		6140 (12.4)		6015 (14.4)		9441 (42.4)		8599 (29.5)	
	Ultra-Orthodox	8527 (2.7)		953 (2.7)		690 (2.0)		1787 (3.6)		1502 (3.6)		378 (1.7)		699 (2.4)	
**SES^b^, n (%)**															
	High	97,556 (31.4)		10,444 (29.2)		10,740 (31.5)		15,873 (32.0)		11,897 (28.5)		6111 (27.4)		7304 (25.0)	
	Medium	126,057 (40.5)		12,458 (34.9)		11,629 (34.1)		22,136 (44.7)		18,920 (45.3)		6016 (27.0)		10,842 (37.1)	
	Low	83,677 (26.9)		12,479 (34.9)		11,238 (33.0)		11,138 (22.5)		10,568 (25.3)		9669 (43.4)		10,158 (34.8)	
	N/A^c^	3878 (1.2)		338 (0.9)		453 (1.3)		393 (0.8)		350 (0.8)		479 (2.2)		893 (3.1)	
**BMI, n (%)**															
	Obese	122,984 (39.5)		10,395 (29.1)		11,453 (33.6)		19,181 (38.7)		17,280 (41.4)		9830 (44.1)		13,210 (45.2)	
	Overweight	107,793 (34.6)		10,125 (28.3)		10,406 (30.6)		17,740 (35.8)		15,549 (37.3)		7845 (35.2)		10,355 (35.5)	
	Normal	47,193 (15.2)		4635 (13.0)		4416 (13.0)		7760 (15.7)		6748 (16.2)		2985 (13.4)		4481 (15.3)	
	Underweight	1255 (0.4)		124 (0.3)		104 (0.3)		192 (0.4)		162 (0.4)		76 (0.3)		146 (0.5)	
	Unavailable	31,943 (10.3)		10,440 (29.2)		7681 (22.6)		4667 (9.4)		1996 (4.8)		1539 (6.9)		1005 (3.4)	
**Smoking status, n (%)**															
	Nonsmoker	136,815 (44.0)		16,116 (45.1)		13,359 (39.2)		21,896 (44.2)		19,062 (45.7)		9622 (43.2)		12,928 (44.3)	
	Past smoker	67,300 (21.6)		6294 (17.6)		6249 (18.3)		10,050 (20.3)		8434 (20.2)		5061 (22.7)		6481 (22.2)	
	Current smoker	43,190 (13.9)		7064 (19.8)		4259 (12.5)		7649 (15.4)		5546 (13.3)		2959 (13.3)		3765 (12.9)	
	Unavailable	63,863 (20.5)		6245 (17.5)		10,193 (29.9)		9945 (20.1)		8693 (20.8)		4633 (20.8)		6023 (20.6)	
ACG^d^, median (IQR)			4 (3-5)		3 (2-4)		3 (3-4)		4 (3-4)		4 (4-5)		4 (4-5)		4 (4-5)
**HbA_1c_^e^ (mmol/mol)**															
	n (%)	27,3491 (87.9)		22,748 (63.3)		27,951 (82.1)		43,651 (88.1)		39,015 (93.5)		20,687 (92.9)		23,249 (93.3)	
	Mean (SD)	7.19 (1.46)		7.31 (1.73)		7.16 (1.39)		7.22 (1.47)		7.15 (1.41)		7.22 (1.45)		7.39 (1.57)	
**Cholesterol (mg/dL)**															
	n (%)	282,583 (90.8)		24,541 (68.7)		29,139 (85.6)		45,069 (91.0)		40,035 (95.9)		21,306 (95.6)		27,832 (95.3)	
	Mean (SD)	167.78 (41.08)		186.01 (43.7)		167.41 (39.5)		169.09 (40.8)		167.25 (40.05)		166.54 (40.01)		164.40 (40.47)	
**Adherence, n (%)**															
	Not treated	65,873 (21.2)		14,213 (39.8)		7078 (20.8)		9126 (18.4)		7246 (17.4)		4004 (18.0)		5475 (18.8)	
	0%	10,501 (3.4)		2601 (7.3)		729 (2.1)		1731 (3.5)		1254 (3.0)		710 (3.2)		792 (2.7)	
	1%-19%	11,990 (3.9)		2141 (6.0)		804 (2.4)		1794 (3.6)		1691 (4.1)		1034 (4.6)		1229 (4.2)	
	20%-39%	17,573 (5.6)		3195 (8.9)		1312 (3.9)		2903 (5.9)		2497 (6.0)		1350 (6.1)		1583 (5.4)	
	40%-59%	27,107 (8.7)		3502 (9.8)		2538 (7.5)		4823 (9.7)		3987 (9.6)		1907 (8.6)		2411 (8.3)	
	60%-79%	31,447 (10.1)		3104 (8.7)		3168 (9.3)		5350 (10.8)		4690 (11.2)		2236 (10.0)		3031 (10.4)	
	≥80%	146,677 (47.1)		6963 (19.5)		18,431 (54.1)		23,813 (48.1)		20,370 (48.8)		11,034 (49.5)		14,676 (50.3)	

^a^IQR: interquartile range.

^b^SES: socioeconomic status.

^c^N/A: not applicable.

^d^ACG: adjusted clinical group.

^e^HbA_1c_: glycated hemoglobin.

**Table 3:** Sociodemographic, clinical, and biological measurements data summary for patients with diabetes who had a moderate-to-high health care services consumption in 2016.

Characteristic		Overall (n=311,168)	High electronic contacts		Automated interaction early adopters (n=26,290)	Human-based contacts		Overall high contact (n=9736)	Electronic driven interaction (n=14,647)
			Internet website (n=19,277)	Smartphone (n=13,279)		Nursing-centered (n=7276)	Physician-centered (n=8137)		
**Gender, n (%)**									
	Female	156,269 (50.2)	8652 (44.9)	7250 (54.6)	15,034 (57.2)	3939 (54.1)	3815 (46.9)	4649 (47.8)	6227 (42.5)
	Male	154,899 (49.8)	10,625 (55.1)	6029 (45.4)	11,256 (42.8)	3337 (45.9)	4322 (53.1)	5087 (52.2)	8420 (57.5)
Age in years, median (IQR^a^)		68 (60-77)	68 (61-75)	64 (56-72)	72 (65-79)	70 (61-79)	73 (65-81)	71 (65-79)	65 (59-71)
**Immigrant, n (%)**									
	No	152,533 (49.0)	8499 (44.1)	6645 (50.0)	9489 (36.1)	3230 (44.4)	3349 (41.2)	3556 (36.5)	7231 (50.6)
	Yes	158,635 (51.0)	10,778 (55.9)	6634 (50.0)	16,801 (63.9)	4046 (55.6)	4788 (58.8)	6180 (63.5)	7416 (50.6)
**Ethnicity, n (%)**									
	General	242,022 (77.8)	18,498 (96.0)	10,977 (82.7)	21,399 (81.4)	5745 (79.0)	6255 (76.9)	9241 (94.9)	14,003 (95.6)
	Arab	60,619 (19.5)	478 (2.5)	2105 (15.9)	3895 (14.8)	1311 (18.0)	1656 (20.4)	313 (3.2)	248 (1.7)
	Ultra-Orthodox	8527 (2.7)	301 (1.6)	197 (1.5)	996 (3.8)	220 (3.0)	226 (2.8)	182 (1.9)	396 (2.7)
**SES^b^, n (%)**									
	High	97,556 (31.4)	9961 (51.7)	3625 (27.3)	7079 (26.9)	1895 (26.0)	2101 (25.8)	4535 (46.6)	5991 (40.9)
	Medium	126,057 (40.5)	7729 (40.1)	5981 (45.0)	12,231 (46.5)	3292 (45.2)	3525 (43.3)	4275 (43.9)	7023 (47.9)
	Low	83,677 (26.9)	1430 (7.4)	3511 (26.4)	6683 (25.4)	2041 (28.1)	2417 (29.7)	842 (8.6)	1503 (10.3)
	N/A^c^	3878 (1.2)	157 (0.8)	162 (1.2)	297 (1.1)	48 (0.7)	94 (1.2)	84 (0.9)	130 (0.9)
**BMI, n (%)**									
	Obese	122,984 (39.5)	7421 (38.5)	6174 (46.5)	12,154 (46.2)	3128 (43.0)	3336 (41.0)	4071 (41.8)	5351 (36.5)
	Overweight	107,793 (34.6)	7299 (37.9)	4512 (34.0)	9374 (35.7)	2514 (34.6)	2800 (34.4)	3749 (38.5)	5525 (37.7)
	Normal	47,193 (15.2)	3123 (16.2)	1819 (13.7)	4316 (16.4)	1307 (18.0)	1621 (19.9)	1666 (17.1)	2316 (15.8)
	Underweight	1255 (0.4)	48 (0.2)	53 (0.4)	112 (0.4)	55 (0.8)	97 (1.2)	42 (0.4)	44 (0.3)
	Unavailable	31,943 (10.3)	1386 (7.2)	721 (5.4)	334 (1.3)	272 (3.7)	283 (3.5)	208 (2.1)	1411 (9.6)
**Smoking status, n (%)**									
	Nonsmoker	136,815 (44.0)	8769 (45.5)	6669 (50.2)	11,955 (45.5)	3034 (41.7)	2951 (36.3)	4131 (42.4)	6323 (43.2)
	Past smoker	67,300 (21.6)	5127 (26.6)	3086 (23.2)	5904 (22.5)	1582 (21.7)	2119 (26.0)	2835 (29.1)	4078 (27.8)
	Current smoker	43,190 (13.9)	1862 (9.7)	1813 (13.7)	2799 (10.6)	1191 (16.4)	1087 (13.4)	756 (7.8)	2440 (16.7)
	Unavailable	63,863 (20.5)	3519 (18.3)	1711 (12.9)	5632 (21.4)	1469 (20.2)	1980 (24.3)	2014 (20.7)	1806 (12.3)
ACG^d^, median (IQR)		4 (3-5)	4 (3-5)	4 (4-5)	5 (4-5)	5 (4-5)	5 (5-6)	5 (4-5)	4 (3-5)
**HbA_1c_^e^, (mmol/mol)**									
	n (%)	273,491 (87.9)	17,810 (92.4)	12,387 (93.3)	25,320 (96.3)	6769 (93.0)	7594 (93.3)	9373 (96.3)	12,936 (88.3)
	Mean (SD)	7.19 (1.46)	6.86 (1.13)	7.19 (1.46)	7.22 (1.42)	7.24 (1.57)	7.26 (1.60)	6.91 (1.17)	7.15 (1.37)
**Cholesterol (mg/dL)**									
	n (%)	282,583 (90.8)	18,399 (95.4)	12,713 (96.0)	25,849 (98.3)	6973 (95.8)	7875 (96.8)	9586 (98.5)	13,232 (80.3)
	Mean (SD)	167.78 (41.08)	165.51 (39.4)	166.77 (40.54)	161.96 (39.28)	169.36 (44.28)	159.30 (44.77)	160.79 (39.47)	165.85 (40.10)
**Adherence, n (%)**									
	Not treated	65,873 (21.2)	4020 (20.9)	2487 (18.7)	4278 (16.3)	1552 (21.3)	2094 (25.7)	1889 (19.4)	2411 (16.5)
	0%	10,501 (3.4)	503 (2.6)	448 (3.4)	617 (2.3)	245 (3.4)	253 (3.1)	239 (2.5)	379 (2.6)
	1%-19%	11,990 (3.9)	458 (2.4)	548 (4.1)	941 (3.6)	357 (4.9)	341 (4.2)	269 (2.8)	383 (2.6)
	20%-39%	17,573 (5.6)	723 (3.8)	823 (6.2)	1346 (5.1)	489 (6.7)	462 (5.7)	346 (3.6)	544 (3.7)
	40%-59%	27,107 (8.7)	1325 (6.9)	1234 (9.3)	2156 (8.2)	697 (9.6)	741 (9.1)	560 (5.8)	1226 (8.4)
	60%-79%	31,447 (10.1)	1711 (8.9)	1447 (10.9)	2712 (10.3)	849 (11.7)	819 (10.1)	804 (8.3)	1526 (10.4)
	≥80%	146,677 (47.1)	10,537 (54.7)	6292 (47.4)	14,240 (54.2)	3087 (42.4)	3427 (42.1)	5629 (57.8)	8178 (55.8)

^a^IQR: interquartile range.

^b^SES: socioeconomic status.

^c^N/A: not applicable.

^d^ACG: adjusted clinical group.

^e^HbA_1c_: glycated hemoglobin.

**Figure 3:**
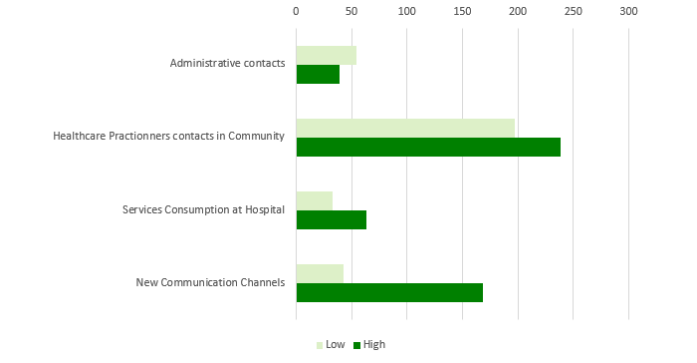
Low-to-moderate versus moderate-to-high health care services consumption of patients with diabetes in 2016.

**Figure 4:**
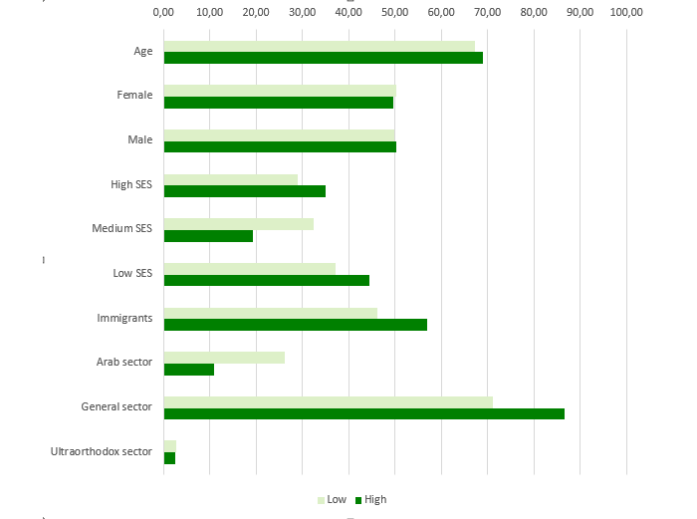
Differences in sociodemographic attributes between low-to-moderate and moderate-to-high health care services consumption of patients with diabetes in 2016. SES: socioeconomic status.

**Figure 5:**
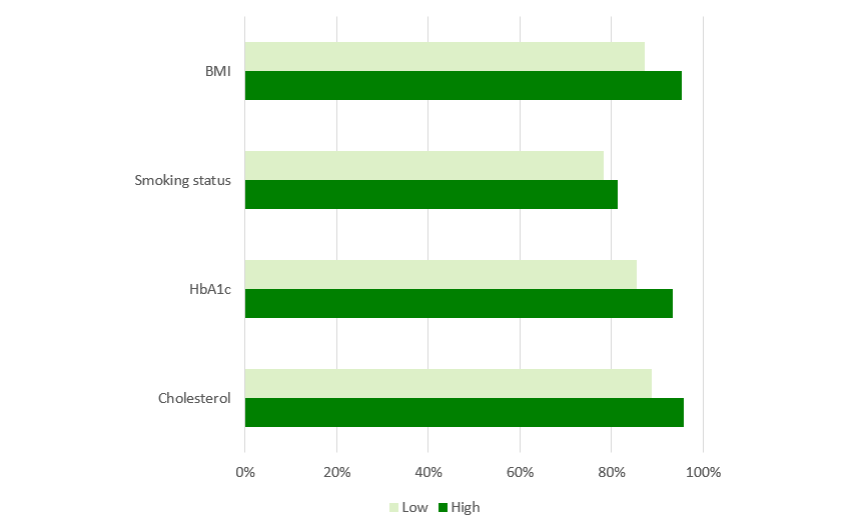
Differences of bioclinical follow-up quality between low-to-moderate and moderate-to-high health care services consumption of patients with diabetes in 2016. HbA1c: glycated hemoglobin.

**Figure 6:**
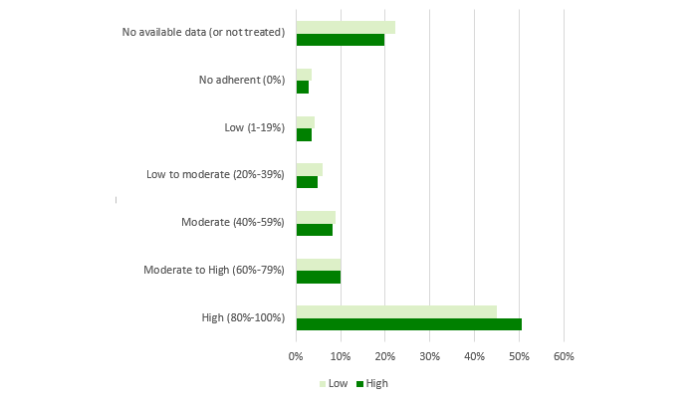
Differences of adherence to treatment between low-to-moderate and moderate-to-high health care services consumption of patients with diabetes in 2016.

Figures 3-6 show charts comparing the differences between the low-to-moderate and moderate-to-high health care services consumer clusters.

Figure 3 shows the differences in health care services use:

- Administrative contacts relate to scheduling an appointment or submitting a nonqueue request (NQR) by a face-to-face meeting at clinic or call to clinic or call center
- Health care practitioner contacts in community relate to physician and nurse consultations and overall visits to pharmacies
- Service consumption at hospital relates to hospitalization and visits to emergency departments
- New communication channels relate to scheduling an appointment or submitting an NQR by using the HMO internet website or smartphone app or answering an SMS suggesting a prescription renewal

Low-to-moderate health care services customers use more administrative contacts than moderate-to-high patients, who use more of the other communication methods.

Figure 4 depicts sociodemographic data. The differences in age and gender are relatively small. There is a higher representation of females in the low-to-moderate cluster, and its patients are slightly younger. More profound differences are at the socioeconomic status (SES) and religious sector. In the low-to-moderate group, there is a higher representation of patients with medium SES, and it has a higher representation of patients from the Arab sector. The moderate-to-high cluster comprises patients with low and high SES mainly from the general (Jewish sector) and immigrant sectors.

Figure 5 compares bioclinical follow-up quality. Generally, the follow-up quality is better in the moderate-to-high cluster than in the low-to-moderate one.

Figure 6 relates to adherence to treatment. A higher proportion of patients from the low-to-moderate cluster are not treated for diabetes, and the adherence of the ones who are treated is much lower than patients in the moderate-to-high cluster.

Generally, low-to-moderate health care services customers tend to prefer direct contacts with health care professionals, and this is probably the cause for lower follow-up quality and adherence to treatment. Conversely, patients in the moderate-to-high clusters use myriad communication channels.

Below we describe the 13 communication profiles found in 2016 and characterize them based on sociodemographic and bioclinical data available. Keeping the population of each one constant, we describe how the communication behavior has changed from 2009 to 2016 as health care professionals introduced new technological means of communications.

### Low-to-Moderate Health Care Services Customers

#### Relatively Low Contact

##### Tech-Based

**Figure 7:**
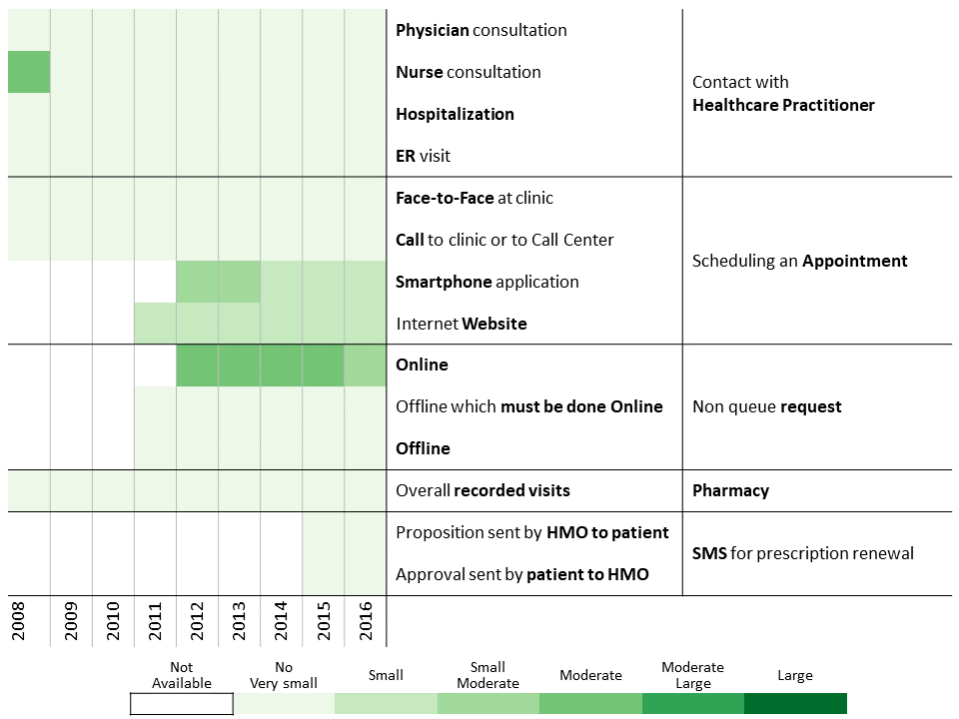
Communication pattern changes between 2008 and 2016 for the relatively low contacts — tech-based group.

This cluster includes 11.48% (35,719/311,168) of the cohort. Patients in this group use fewer physical interactions and tend to be early adopters of new channels (Table 2 and Figure 7). They exhibit a relatively high use of electronic channels for scheduling appointments and online NQR tools when these channels became available. They are relatively young, men are highly represented (20,039/35,719, 56.10%), as are the Arab sector (10,537/35,719, 29.49%) and low SES population (12,479/35,719, 34.93%). Their follow-up quality is relatively poor but progressively improving. The missing measurements of BMI (2008: 37.30% [13,323/35,719]; 2016: 29.20% [10,430/35,719]) and HbA_1c_ (2008: 56.30% [20,110/35,719]; 2016: 36.30% [12,966/35,719]) decreased over time. Despite the aging, the HbA_1c_ average increased just a little (7.06 [SD 1.58] mmol/mol vs 7.31 [SD 1.7] mmol/mol). Of patients who were followed up, the percentage of patients treated for diabetes increased (2009: 32.8% [11,716/35,719]; 2016: 60.20% [21,503/35,719]), as did the proportion of highly adherent patients (2009: 13.40% [4786/35,719], 2016: 19.50% [6965/35,719]), with significant changes in 2011 and 2012 when NQRs and online (website and smartphone app) appointment scheduling were introduced. This group also started using the SMS channel for renewing prescriptions in 2015 when this channel was launched.

##### Non–Clinic-Based

**Figure 8:**
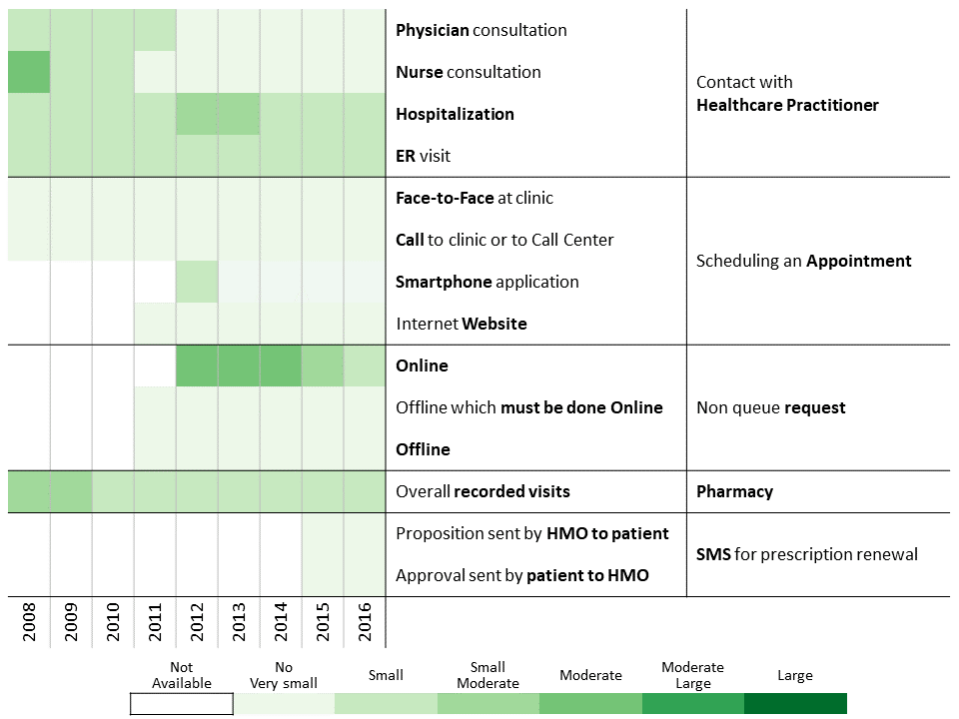
Communication pattern changes between 2008 and 2016 for the relatively low contacts — non–clinic-based group.

This cluster includes 10.95% of the cohort (34,060/311,168; Figure 8). Its median age is similar to the cohort, and the Arab sector is highly represented (9884/34,060, 29.02%). Until 2011, communication between patients and health care professionals was mainly achieved directly with the health care professionals. When electronic channels became available that year, the proportion of visits to health care professionals decreased in favor of online NQRs. The proportion of missing follow-up measurements is stable at around 20% each year. HbA_1c_ values are also relatively stable over time (7.16 [SD 1.39] mmol/mol in 2016). The percentage of patients treated for diabetes increased (2009: 56.50% [19,244/34,060]; 2016: 78.20% [26,635/34,060]) and is associated with an increase in high proportion of days covered (2009: 36.01% [12,266/34,060]; 2016: 54.11% [18,431/34,060]). This improvement in adherence over time is correlated with the use of the different electronic nonclinic contacts.

##### Tech-Based Versus Non–Clinic-Based

Analyzing the relatively low contact groups over time reveals that while the tech-based group prefers technology-based means of communication, the non–clinic-based group has a relatively high number of hospitalizations and visits to the emergency department compared with the rest of the population.

While the introduction of new digital communication channels is correlated with an increase in adherence to treatment for the tech-based group, patients in the non–clinic-based group do not exhibit this kind of behavior. On the contrary, there is a correlation between the introduction of new communication channels and a reduction in the average number of physician visits. For the tech-based cluster, the introduction of new communication channels might be viewed as an opportunity to decrease the number of contacts with the HMO.

#### Low and Scheduled Visits

##### Moderate Nonvisits

**Figure 9:**
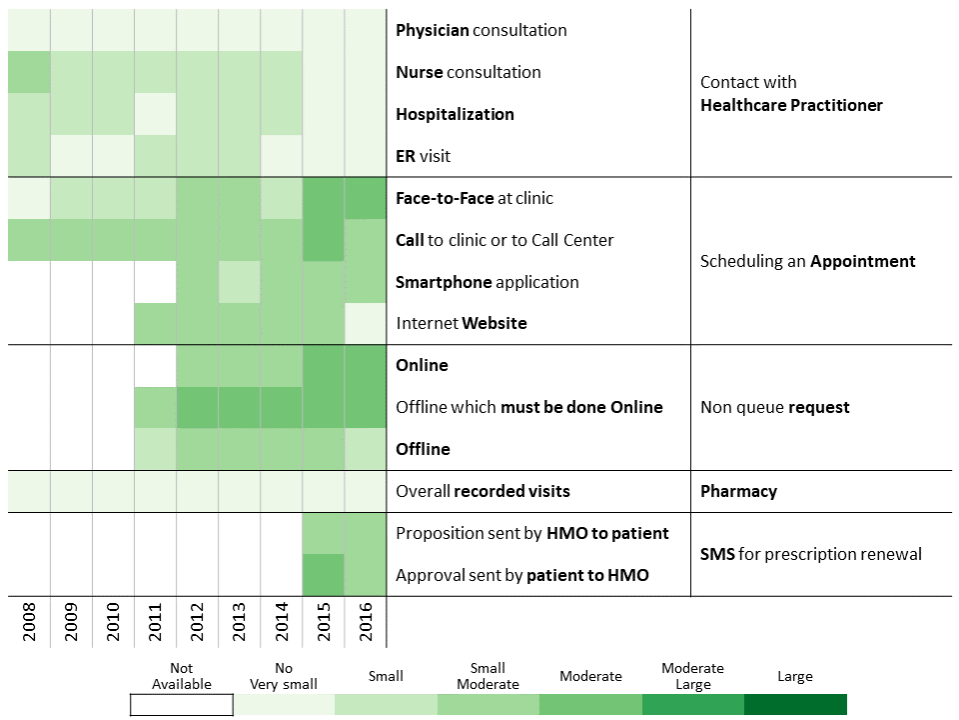
Communication patterns for patients having a low number but scheduled visits and preferring communication channels that do not involve a face-to-face meeting.

This group, which includes 15.92% (49,540/311,168) of the cohort, comprises patients who mainly use communication channels that do not involve face-to-face consultations (Table 2 and Figure 9). Its median age is close to the cohort median age of 67 years, and the general sector is highly represented (41,613/49,540, 84.00%). Until 2011 when the first electronic channels were introduced, patients in this group resorted to the available communication channels apart from physicians such as consulting nurses, hospitalizations, and emergency department visits. Once electronic options were introduced, the volume of contacts with health care professionals decreased in favor of the online tools for scheduling appointments and NQRs. Furthermore, this group was proactive and had a high answering rate to the SMS for automated prescription renewal. The proportion of patients taking a medication for diabetes jumped from 51.40% (25,464/49,540) in 2009 to 81.60% (40,425/49,540) in 2016. These values are associated with a high adherence to treatment, which increased by 17.70% between 2009 and 2016. Even though these patients consulted health care professionals less often, the proportion of missing annual measurements of follow-up metrics dropped from more than 20% in 2008 to approximatively 10% in 2016. For this subpopulation, additional channels are an opportunity to adjust communication patterns with the HMO to their preferences.

##### Low Balance Use

**Figure 10:**
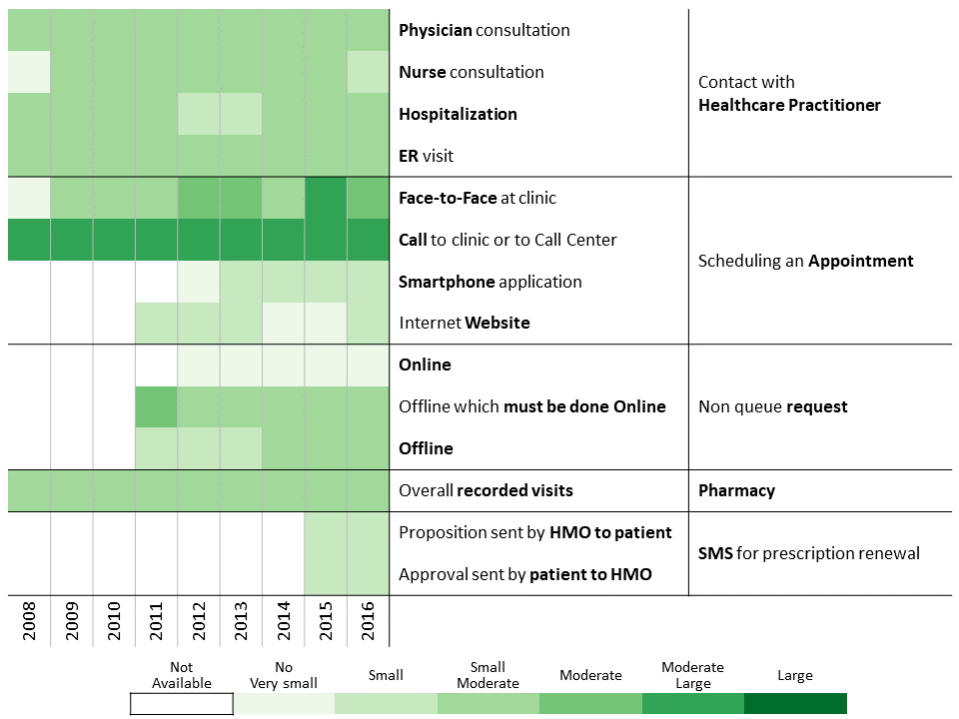
Communication patterns for patients with diabetes having low balance use of health care services and scheduled visits.

This cluster includes 13.41% (41,735/311,168) of the cohort (Table 2 and Figure 10). These patients are using all available services at a relatively low rate. The cluster median age is higher than the cohort, and there is a higher proportion of immigrants. Despite the diversity of available and newly introduced communication channels, health care services consumption is stable. These patients prefer traditional channels for appointment scheduling (ie, face-to-face at clinic, call to clinic or call center). Adding technology-based channels has a marginal effect on communication with health care professionals. Clinically, adjusted clinical group (ACG) level remains relatively stable over time (2010: 4 [3;4]; 2016: 4 [4;5]), and HbA_1c_ level is globally controlled (2016: 7.15 [SD 1.41] mmol/mol). Despite low use of available channels, patients have high rates of adherence to follow-up and treatment that increase over time. The proportion of missing annual measurements of follow-up metrics dropped (2008: around 15%; 2016: around 7%), and the percentage of patients with high adherence increased. This change may be attributed to aging and changes in therapeutic status (patients taking a noninsulin medication for diabetes jumped from 51.40% [25,464/49,540] in 2009 to 81.60% [40,425/49,540] in 2016).

#### Measured Human Contacts

Measured human contacts means a relatively low use of appointment scheduling, NQRs, and SMS channels. Despite relatively low human contacts, these patients exhibit a moderate level of consulting health care professionals. Their median comorbidity level is relatively stable over time (2010: 4 [3;5]; 2016: 4 [4;5]) in view of the population aging.

##### Nonplanners

**Figure 11:**
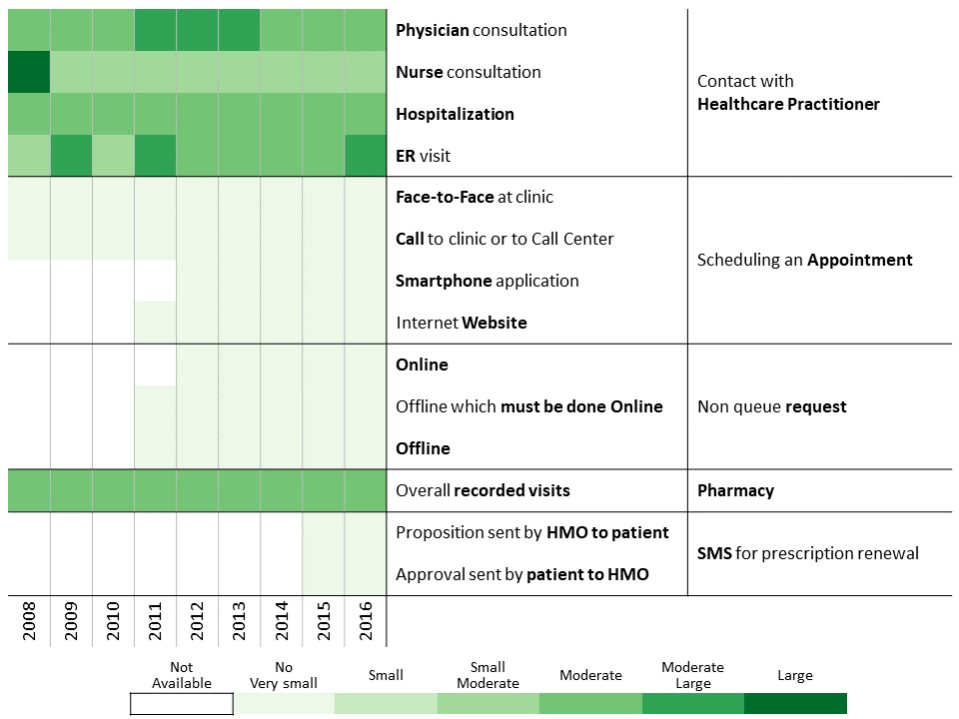
Communication patterns for patients having measured human contacts without generally scheduling their visits.

Nonplanners constitute 7.16% (22,275/311,168) of the cohort. They have a relatively moderate-to-high human health care services consumption but a low tendency to use nonhuman means of communication (Table 2 and Figure 11). This group consists of a larger proportion of patients from the Arab sector (9441/22,275, 42.38%), which is associated with a higher proportion of nonimmigrants (14,504/22,275, 65.11%) and people with low SES level (9669/22,275, 43.40%). The preference of nonplanned human contact does not have a negative clinical effect. HbA_1c_ values of the nonplanners are controlled over time (2010: 7.26 [SD 1.52] mmol/mol; 2016: 7.22 [SD 1.45] mmol/mol) with very good follow-up and adherence levels. The proportion of missing follow-up measurements dropped over time, and this improvement is associated with an increased number of patients treated with diabetes medication (2010: 61.51% [13,701/22,275]; 2016: 82.02% [18,271/22,275]) and better adherence over time (2010: 36.35% [8097/22,275]; 2016: 49.54% [11,034/22,275]).

##### Nursing Contacts

**Figure 12:**
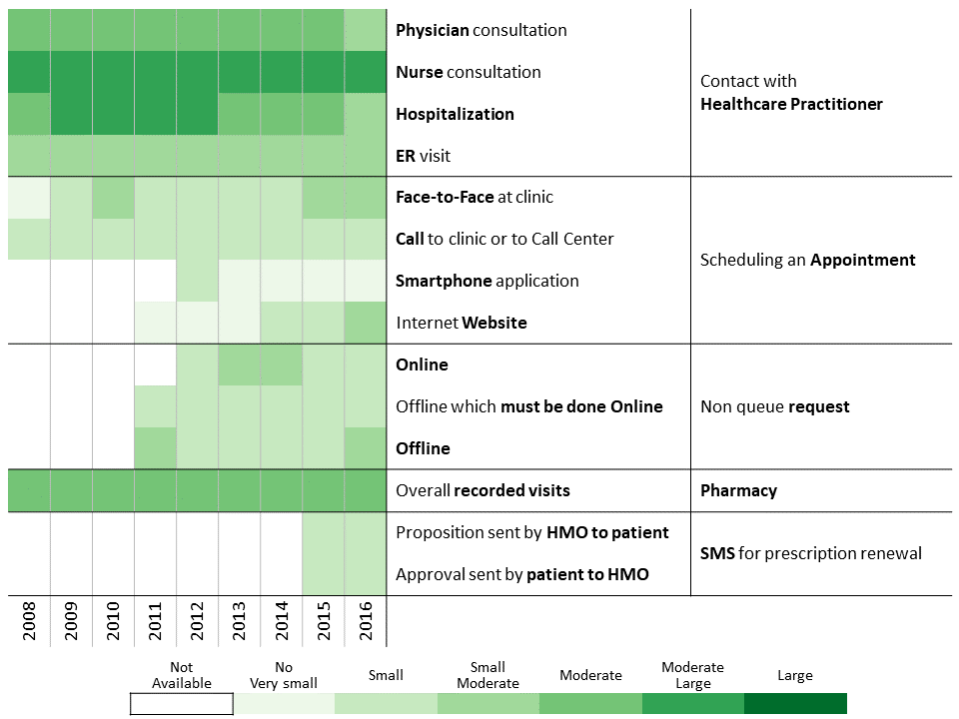
Communication pattern changes for patients with diabetes having measured human contacts mainly by consulting nurses.

The nursing-based contacts cluster constitutes 9.38% (29,197/311,168) of the cohort (Table 2 and Figure 12), and its members prefer to consult with nurses. They also tend to schedule their appointments by using all available channels. The proportion of females is slightly higher than in the cohort (15,463/29,197, 52.99%), as are the proportions of nonimmigrant patients (16,085/29,197, 55.09%), Arab sector representation (8599/29,197, 29.45%), and low SES population (10,158/29,197, 34.79%). These demographics may explain the tendency to rely on contacts with health care professionals (human contacts). The average HbA_1c_ level is stable over time (2010: 7.42 [SD 1.60] mmol/mol; 2016: 7.39 [SD 1.57] mmol/mol) and higher than the overall cohort. Although these results show merely a correlation between use of the nurse-patient channel and high levels of follow-up and adherence, they raise the hypothesis that the nurse-patient channel is very effective in inducing follow-up and adherence levels of patients. The percentage of patients who missed their annual measurements of follow-up metrics dropped over time (2008: around 15%, 2016: around 4%) and the adherence level increased (2010: 42.07% [12,284/29,197]; 2016: 50.27% [14,676/29,197]), as did the proportion of patients receiving treatment for diabetes (2010: 65.90% [19,240/29,197]; 2016: 81.25% [23,722/29,197]).

#### High Electronic Contacts

**Figure 13:**
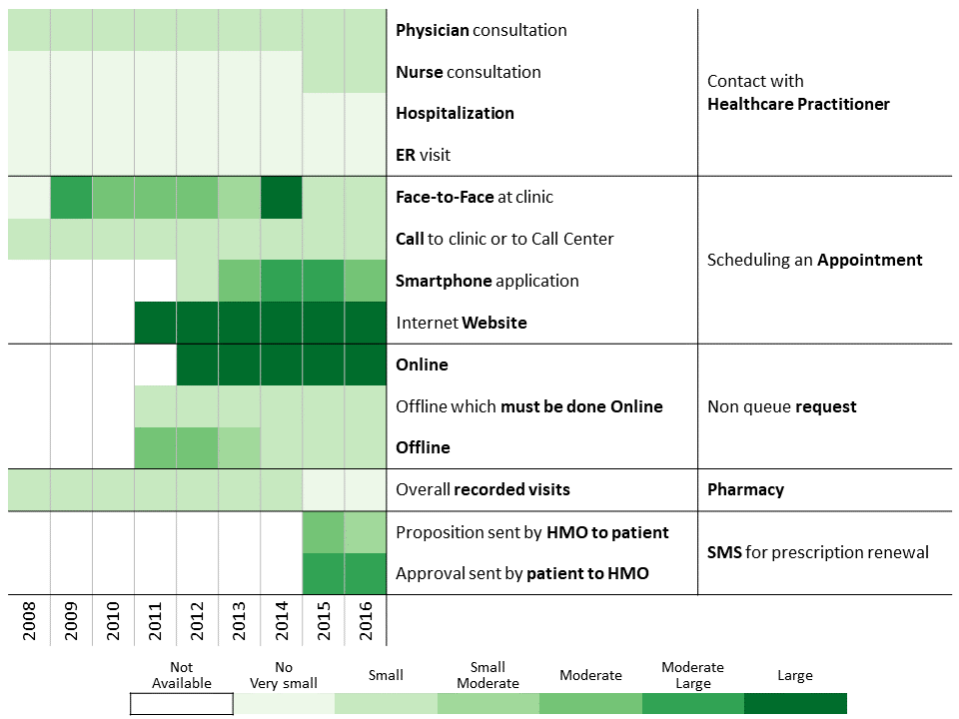
Communication pattern changes for patients having a high volume of electronic contacts over the health management organization website.

**Figure 14:**
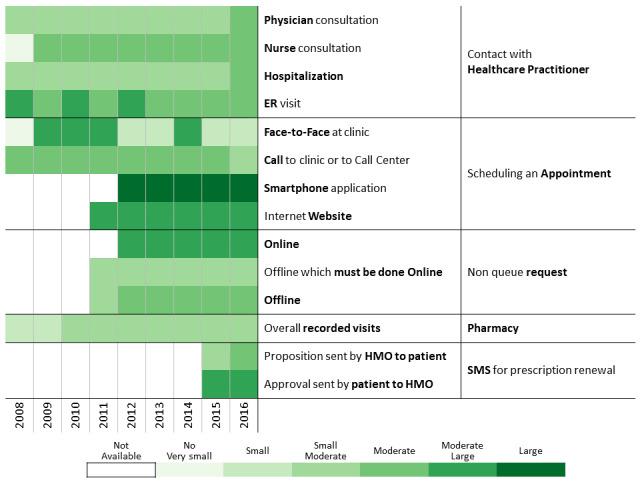
Communication pattern changes between 2008 and 2016 for patients with diabetes having a high volume of electronic contacts over the health management organization smartphone app.

Patients in these clusters have a high level of use of online and electronic channels for communicating with the HMO (Table 3, Figure 13, and Figure 14). The website group (those using a personal computer) comprises 6.20% of the cohort (19,277/311,168) and the smartphone (those using a mobile app) group comprises 4.27% (13,279/311,168).

##### Differences Between Website and Smartphone Clusters

These patients exhibit substantial use of the most prominent technological interfaces developed in the past 20 years, with smartphone users being younger. This phenomenon might relate to the lower penetration rate of new technologies in older populations. The gender profiles of the two groups differ (website: males, 55.12% [10,625/19,277]; smartphone: females: 54.60%, [7250/13,279]). Moreover, in both groups (website/internet and smartphone users) there is a higher representation of the general population (respectively 95.96% [18,498/19,277] and 82.66% [10,977/13,279] vs the overall population 77.8% (242,022/311,168]) and a higher representation of medium and high SES (respectively, the website/internet users having a high SES are 51.67% [9961/19,277] and 45.04% [5981/13,279] and the people of the smartphone cluster have a medium SES). This observation conforms to prior research, which found that lower SES populations gravitate toward smartphones [60,61].

##### Common Findings Between Website and Smartphone Clusters

The heatmaps (Figure 13 and Figure 14) show high use of the website and smartphone app. Nevertheless, it is possible to see that the website cluster has a relatively low volume of direct contacts with health care professionals and prefers tools that enable nondirect and distant contacts. The clinical follow-up of these two clusters is better than that of the overall population, the proportion of missing follow-up indicators being lower in 2016 (Table 3). Furthermore, treatment adherence was better in 2016 than in the cohort population and also increased over the years in parallel with the number of newly treated patients. Patients in these two clusters tend to reduce their use of other means of communication and contacts with the HMO in favor of electronic media while maintaining a high follow-up quality and treatment adherence. By considering what seems like a positive impact of smartphone presence, the HMO should incorporate more functions into the smartphone app.

#### Automated Interaction Early Adopters

**Figure 15:**
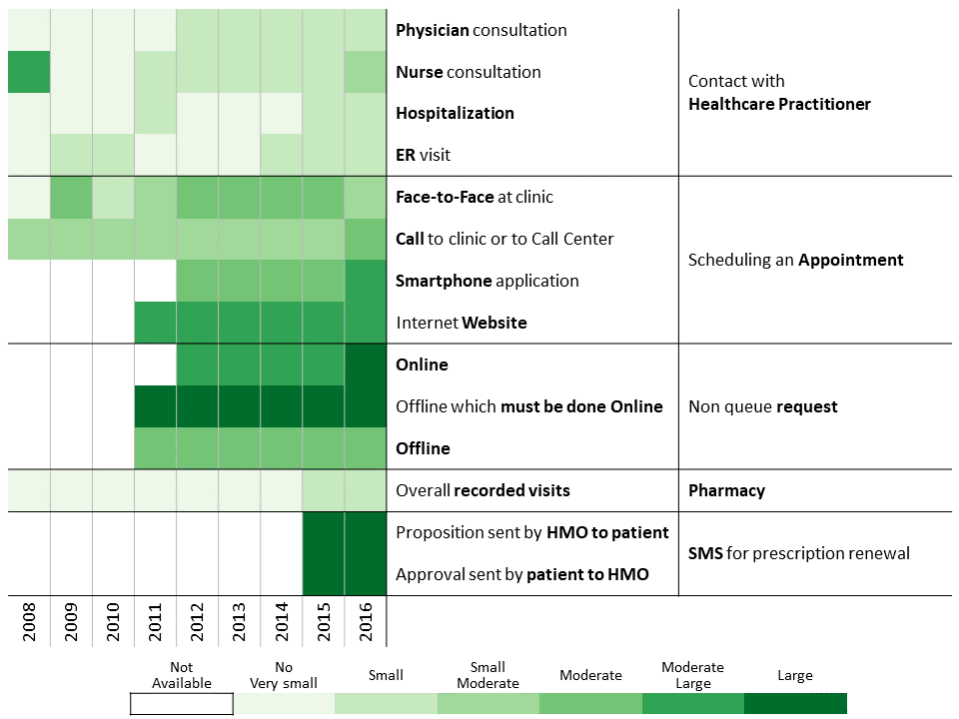
Communication pattern changes between 2008 and 2016 for patients with diabetes being early adopters of automated interaction tools.

These patients are relatively young (Table 3 and Figure 15), and females and the general sector are largely represented (15,034/26,290, 57.19%, and 19,310/26,290, 73.45%, respectively). Despite being early adopters of new interaction services, this cluster uses all human contact–based services over time. The new automated interaction tools improve the quality of contacts with the HMO and do not serve as a replacement to previously existing channels. The comorbidity of this relatively young group is high (ACG: 5 [4;5]), and the proportion of missing bioclinical and follow-up measurements in 2016 is relatively low. Considering the aging process and the diabetes treatment policy change (lowering the HbA_1c_ threshold from 7.5% to 6.5%), the number of patients taking a noninsulin treatment for diabetes increased over time (2010: 66.81% [17,565/26,290]; 2016: 83.73% [22,012/26,290]). Moreover, the proportion of missing follow-up measurements decreased (eg, BMI: 2010, 6.83% [1796/26,290]; 2016, 1.27% [334/26,290]; HbA_1c_, 2010, 17.08% [4492/26,290]; 2016, 3.69% [970/26,290]) and the proportion of adherence to treatment increased (2010: 44.85% [11,792/26,290]; 2016: 54.17% [14,240/26,290] in 2016). These results may indicate that the new tools allow patients to improve their engagement with the HMO. As their disease progresses, an increase in their services consumption is expected, but instead we observe a slight decrease in some of the services consumed. One contributing factor to the high prevalence of patients from the general sector in the cluster may be related to the sole use of Hebrew in the internet and smartphone app.

#### Human-Based Contacts

##### Differences Between Nursing-Centered and Physician-Centered Clusters

**Figure 16:**
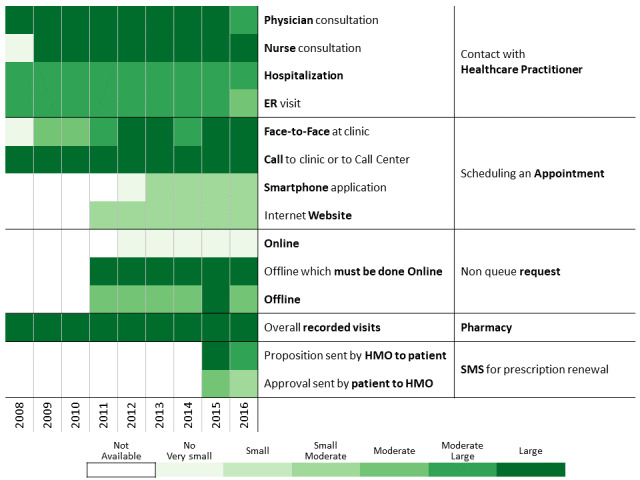
Communication pattern changes between 2008 and 2016 for patients with diabetes having mainly human-based contacts based on interactions with nursing.

**Figure 17:**
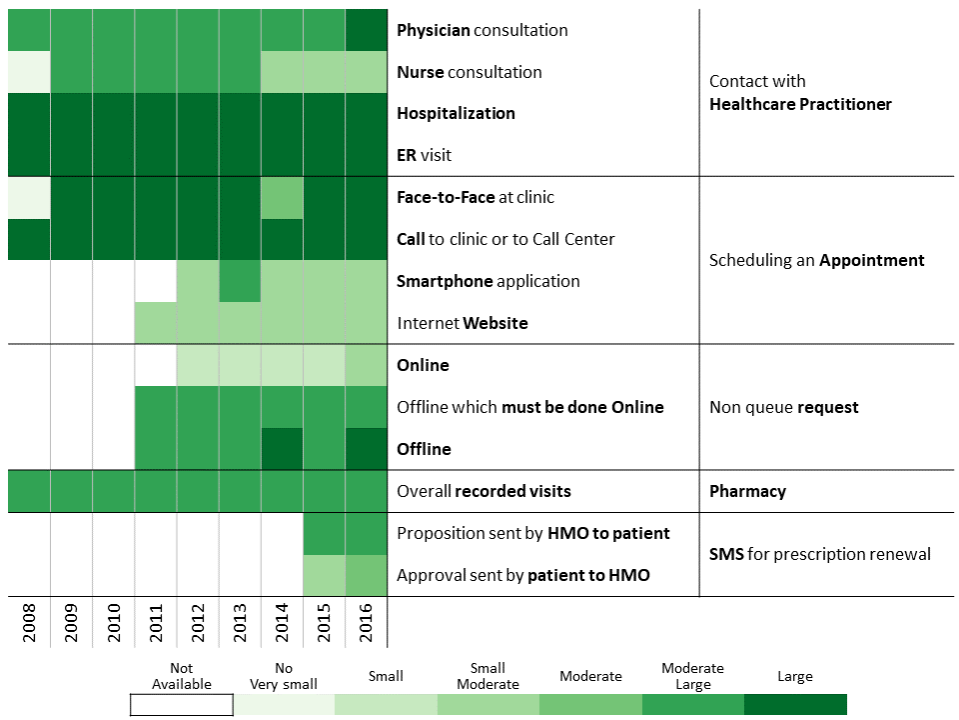
Communication pattern changes between 2008 and 2016 for patients with diabetes having mainly human-based contacts based on interactions with physicians.

Patients having mainly human-based contacts (Table 3) with the HMO are divided in two clusters: nursing-centered (7276/311,168, 2.34%; Figure 16) and physician-centered (8137/311,168, 2.61%; Figure 17). It should be noted that one of the main differences between these two populations is the proportion of patients missing their follow-up measurements in the physician-centered group. This highlights the importance of nurse involvement in patient follow-up.

##### Common Findings in Nursing-Centered and Physician-Centered Clusters

These two clusters are relatively similar. In 2016, the proportion of female patients is relatively high and the population is older, with a majority of immigrants, and a higher representation of patients from medium-low SES groups. Patients in these two clusters had a relatively high ACG score over time. This increasing level of comorbidity can justify the high volume of nurse and physician consultations and high follow-up quality scores.

#### Higher Resource Consumers Having Overall High Contacts and Electronic Driven Interactions

**Figure 18:**
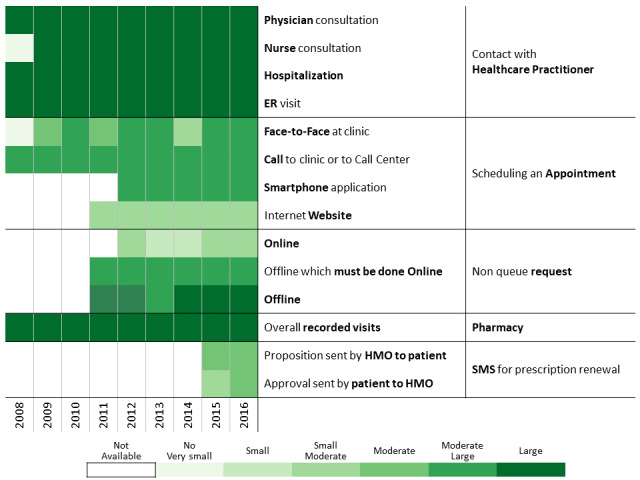
Communication pattern changes between 2008 and 2016 for patients with diabetes having an overall high number of contacts with health care services.

**Figure 19:**
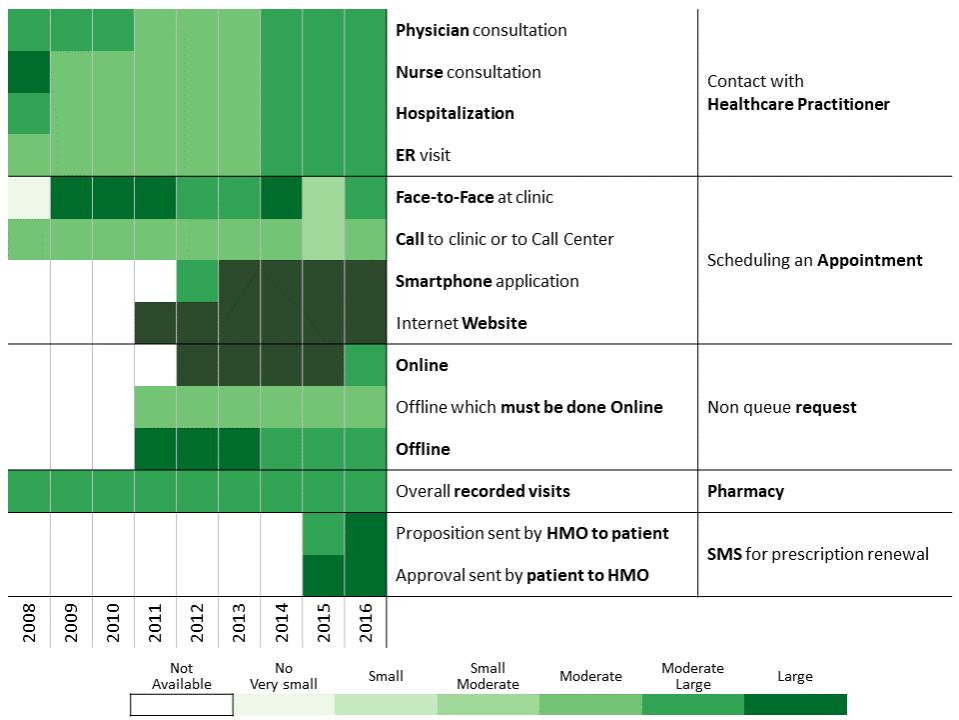
Communication pattern changes between 2008 and 2016 for patients with diabetes leading electronic-driven interaction with the health management organization.

Overall high contact represents 3.13% (9736/311,168) of the cohort (Table 3 and Figure 18) and the electronic driven interaction 4.71% (14,647/311,168; Table 3 and Figure 19). They comprise older people (mean age 71 years) and a higher proportion of men and immigrants. From ethnicity and SES perspectives, the distributions for the overall high contact group resemble the cohort, while the electronic driven interaction group has a higher representation of the general sector (9241/9736, 94.92%) and patients with high SES (4535/9736, 46.58%).

The main differences in the communicational profiles are in the interaction strength. The volume of contacts is high in both groups but higher for the overall high contact group compared with the electronic driven interaction group. This can be explained by the lower SES in the first cluster and Hebrew-speaking abilities, which may be lower than in the second. Lower SES may be considered as a proxy for confidence and ability to use technologies, and immigrant status, age, and SES as proxies for defining language abilities. The introduction of new communication channels over time only increased the global number of contacts. The large number of visits at the clinic may be justified by the higher comorbidity level. Follow-up measurements in both clusters are consistently better than the cohort. Treatment compliance is better for the electronic driven interaction cluster and increasing over time and in parallel to new communication channels introduction. Adherence to treatment of the overall high contact group is around 42% over time and not influenced by the addition of technological channels.

The age and comorbidity levels in these two clusters strongly influence the number of contacts with health care professionals over time and, as a by-product, the quality of follow-up improves. However, results suggest that SES and immigrant status influence the use of new technologies and increase the number of contacts with the HMO and patient adherence to treatment.

## Discussion

### Principal Findings

In this study, we identified and characterized 13 media profiles of patients. We have shown how communication behavior is influenced by the means of communication that the health organization provides to the patient. Additionally we have pointed out how different patients respond to technology-based communication and change the way they communicate with the health organization. Finally, we highlighted that some patients prefer to communicate with the organization by technological means and respond adequately to text messages, others prefer to communicate with the physician, and others with the nurse.

Identifying the channels of communication with the health organization and health care professionals preferred by each patient creates an opportunity to convey messages adapted to the patient in the most suitable communication channel. The greater the likelihood that the therapeutic message is received by the patient, the greater the patient’s response to treatment, and the better the health of the patient.

### Strengths and Limitations

Clalit insures and provides medical services to more than 54% of the Israeli population. It is the largest health care organization and insurer in Israel. However, although Clalit covers most of the population, the overall ethnic distribution of its health care customers does not accurately reflect the Israeli demographic composition: it has a higher proportion of Arabs, a lower proportion of ultra-Orthodox, and a higher proportion of members with a low SES [19].

#### Patients With Diabetes and Generalization to the Overall Chronic Patient Population

This retrospective analysis looks at Israeli patients treated by an Israeli HMO. The Israeli health care system, culture, and norms are factors affecting patient behavior in a specific way that do not allow a direct generalization of the results to other parts of the world.

This study overcomes a limitation of prior research dealing with the identification and description of health care customer communication patterns among individuals with diabetes in Clalit in 2015 [62]. Analyzing data that spans 9 years provides a better understanding of the changes of communication channel use over time and impact of socioeconomic factors, which cannot be easily and clearly understood with a 1-year snapshot.

#### Effect of Digital Communication Tools

The digital tools introduced between 2009 and 2016 for patients diagnosed with diabetes influence their follow-up and communication pathways with health care professionals. However, for more than half of the population investigated in this research, we found only a negligible influence of the digital tools on the communicational behavior (relatively low contacts, low and scheduled visits–low balance uses, measured human contacts, human-based contacts, and overall high contact clusters, 54.30%).

Digital tools, such as NQRs and SMS for prescription renewal, allow patients to reduce or avoid visits to the clinic or hospital. For patients initially having a relatively low number of health care practitioner contacts over time, these digital tools may induce a reduction in the number of visits to the clinic or nurse station. Eliminating potential visits due to the introduction of digital tools might influence the follow-up quality because these visits could have served as another opportunity for a human contact with the patient (eg, for discussing treatment issues) or at least to measure the patient’s condition [61,62]. Almost a third of the research population use technology to reduce their engagement with health care professionals. Not surprisingly, these are patients who tend to have a relatively small number of interactions with health care professionals (relatively low contacts–tech-based, low and scheduled visits–moderate nonvisits, electronic users, and automated interaction early adopters, 32.10%). We would like to emphasize that the results do not show that the introduction of digital tools deteriorates the health condition of this one-third of the population. Nor do we claim that the reduction in visits to health care professionals is inherently an unwanted outcome. On the contrary, this is exactly what EMR systems are designed for. Rather, we claim that for targeted populations, which do not communicate efficiently with health care professionals, new digital tools might have negative consequences on the quality of the follow-up. This danger can be mitigated by using additional, human-based communication channels, akin to the guided-care approach [63], which have already proven to be effective. We can now build tools to identify these patients based on their behavior and target the efforts on the population that needs it.

For about 13.60% of the population, the introduction of new and digital channels in the communication arsenal of the HMO is effective. These are the patients who belong to the following clusters: high electronic contacts–website, high electronic contacts–smartphone, and electronic driven interaction.

Patients in the low-to-moderate clusters were found to have different health outcomes due to a lower health care services consumption impacting their follow-up quality and adherence to treatment. On the other hand, patients who consume more services, the ones in the moderate-to-high clusters, have better health outcomes (despite being generally older and with a higher ACG). To sum up, it looks like that the effect of digital communication tools is to improve the follow-up and adherence to treatment instead of replacing human interactions with health care professionals.

### Current and Potential Future Directions

As time progresses, the population becomes more accustomed to using digital channels and new communication channels are introduced (eg, an online counseling services with video calls to physicians when clinics are closed, available in Clalit since 2017). Communication patterns should be monitored in the face of the rapid changes in population behavior and services offered.

Furthermore, by tuning its communication tools to patient preferences and special needs (eg, by translating the user interfaces of electronic communications tools to languages such as Arabic, English, Russian, Amharic, French, Spanish), the health organization would realize the following:

- Improve and increase accessibility to health care services, achieve better patient engagement and responsiveness to treatment, and improve quality of treatment and treatment experience within existing budgetary constraints
- Increase patient engagement with the treatment process by transforming the communication scheme with each patient to a more proactive scheme to better fit patient profile
- Allow patient-reported outcome measures [64] for some follow-up measurements such as BMI (or more specifically, weight) and smoking status in an effort to reduce nurse work (over)load while continuing and improving patient follow-up

Finally, we investigated only diabetic patients. This research and its related methodology can be generalized and extended to other chronic and acute patients.

### Conclusion

In this paper we presented and demonstrated a methodology to identify communication profiles over time within health care systems. We applied this methodology to the data of more than 300,000 diabetic patients from Clalit Health Services in Israel and found 13 such profiles. These profiles enabled health care professionals and the insurer to adapt the communication and message conveyed to patients based on their communication profile. This methodology can be applied in other organizations in other geographical locations.

We found that 22.40% of patients have very low health services consumption, and an additional 45.90% have low-to-moderate health services consumption, which indicated a low level of patient engagement. We showed that the introduction of technological communication channels didn’t substantially improve the engagement of these patients and for some of the patients it even reduced communication with health care professionals. Based on these findings, we think that improving patient engagement cannot rely solely on technological solutions; rather, these solutions must be accompanied by complementary means [65].

## Abbreviations

ACG: adjusted clinical groups
Clalit: Clalit Health Services
CRI: Clalit Research Institute
DWH: data warehouse
EMR: electronic medical record
ER: emergency department
HbA_1c_: glycated hemoglobin
HMO: health management organization
KDD: knowledge discovery in databases
NQR: nonqueue request
SES: socioeconomic status
PDC: proportion of days covered
SMS: short message service
